# Preventing the mental health consequences of war in refugee populations

**DOI:** 10.1017/S2045796022000154

**Published:** 2022-04-19

**Authors:** Corrado Barbui, Marianna Purgato, Ceren Acarturk, Rachel Churchill, Pim Cuijpers, Markus Koesters, Marit Sijbrandij, Maritta Välimäki, Johannes Wancata, Ross G. White

**Affiliations:** 1 WHO Collaborating Centre for Research and Training in Mental Health and Service Evaluation, Department of Neuroscience, Biomedicine and Movement Sciences, University of Verona, Verona, Italy; 2 Department of Psychology, College of Social Sciences and Humanities, Koc University, Istanbul, Turkey; 3 University of York, York, UK; 4 Department of Clinical, Neuro, and Developmental Psychology, Amsterdam Public Health Institute, and WHO Collaborating Centre for Research and Dissemination of Psychological Interventions, Vrije Universiteit Amsterdam, Amsterdam, The Netherlands; 5 Department of Psychiatry II, Ulm University, Ulm, Germany; 6 Department of Nursing Science, University of Turku, Turku, Finland; 7 Clinical Division of Social Psychiatry, Department of Psychiatry and Psychotherapy, Medical University of Vienna, Vienna, Austria; 8 School of Psychology, Queen's University Belfast, Belfast, Northern Ireland

## Abstract

The refugee experience is associated with several potentially traumatic events that increase the risk of developing mental health consequences, including worsening of subjective wellbeing and quality of life, and risk of developing mental disorders. Here we present actions that countries hosting forcibly displaced refugees may implement to decrease exposure to potentially traumatic stressors, enhance subjective wellbeing and prevent the onset of mental disorders. A first set of actions refers to the development of reception conditions aiming to decrease exposure to post-migration stressors, and a second set of actions refers to the implementation of evidence-based psychological interventions aimed at reducing stress, preventing the development of mental disorders and enhancing subjective wellbeing.

According to recent UNHCR figures, in 2 weeks over two million refugees were forcibly displaced from Ukraine to neighbouring countries (UNHCR, 2022). This population adds to over 80 million forcibly displaced people worldwide, including over 30 million refugees and asylum seekers (UNHCR, 2022).

The displacement and refugee experience are typically associated with loss of homes, hopes, possessions and disruption of personal, family and professional life projects. In addition, several potentially traumatic events may occur before migration, such as bombings, threats, captivity, torture, injury and witnessing death or injury of loved ones (IASC, 2007). Forcibly displaced people are also exposed to many stressors during migration and post migration. Major threats after arrival in host countries include discrimination, economic problems, language barriers, loss of family and community support, poor access to social, educational and health services, and uncertain asylum application procedures (Sijbrandij, 2018; Jannesari *et al*., 2020).

Owing to exposure to potentially traumatic events and other stressors and ongoing living difficulties, forcibly displaced refugees are at risk of developing mental health consequences, including worsening of subjective wellbeing and quality of life (Beiser and Hou, 2017; van der Boor *et al*., 2020), and risk of developing mental disorders. Blackmore and colleagues, who conducted a systematic review and meta-analysis of prevalence studies in this population, found a prevalence of 30% for depression and post-traumatic stress disorder (PTSD), 11% for anxiety disorders and 1.5% for psychotic disorders (Blackmore *et al*., 2020). Similar figures were obtained by Henkelmann and colleagues for PTSD, depression and anxiety disorders (Henkelmann *et al*., 2020). For psychosis, Brandt and colleagues, who reviewed nine studies involving 540 000 refugees, calculated that the average incidence of psychosis was 43% higher in refugees compared with the nonrefugee population (Brandt *et al*., 2019). Other systematic reviews consolidated these findings by showing that rates of mental disorders such as anxiety, depression and complex PTSD were significantly higher in specific groups of adult refugees (Syrian refugees, torture survivors, refugees resettled in specific countries) than the rates in the general population (Turrini *et al*., 2017; Abu Suhaiban *et al*., 2019; Hoell *et al*., 2021; Mellor *et al*., 2021; Nguyen *et al*., 2022).

Against this background, it is imperative that countries hosting forcibly displaced refugees implement targeted actions aiming to decrease exposure to potentially traumatic stressors, enhance subjective wellbeing and prevent the onset of mental disorders.

A first set of actions refers to the implementation of policies aiming to decrease post-migration stressors, such as material and economic hardship that could affect integrity, independence, dignity and well-being, social hardship due to loss of status, feelings of inadequacy in relation with specific skills needed in the host-country, experiences of unfair treatment on the basis of prejudice or social exclusion. This may be achieved by organising reception conditions optimising internationally recognised minimal quality standards. Standards for the reception of applicants for international protection have been established by Directive 2013/33/EU of the European Parliament (European Union, 2013). The Directive clearly reports that national authorities should ensure that reception modalities are specifically designed to meet the needs of persons requiring international protection, including legal assistance, document provision, material support, links with local communities, freedom of movement, information about labour market access, vocational training, social support. Health care, including mental health care, is also mentioned as a key intervention where needed. It is important to ensure that efforts to support forcibly displaced people are coordinated across the different layers of the social environments in which they are hosted, i.e. at the level of the individual, their family, the community, and the institutions that have governance responsibility for their care and support (White and Van der Boor, 2021). Optimising these factors and harmonising the reception conditions within and across countries may contribute to reduce exposure to potentially traumatic post-migration stressors, therefore decreasing the risk of developing a mental disorder.

In addition to risk reduction policies, national authorities should be aware that evidence-based psychological interventions aimed at reducing stress, preventing the development of mental disorders and enhancing subjective wellbeing are available and may be implemented. As the provision of preventative psychological interventions to the whole population of refugees may not be sustainable by host countries, such interventions may be offered to population groups exposed to specific risk factors associated with increased levels of psychological symptoms. For example, populations at increased risk are those recently resettled in host countries, those living in shared asylum accommodations, people separated from the family, those with uncertain asylum status, and people showing high levels of psychological distress such as symptoms of anxiety, depression or posttraumatic stress (Gleeson *et al*., 2020; Hajak *et al*., 2021). Forcibly displaced refugees with these characteristics should be offered focused psychological interventions (IASC, 2007).

In recent years, the World Health Organization (WHO) has developed a number of low-intensity psychological interventions that may be scaled up as public health strategies to provide psychological support to refugee populations exposed to adversities (WHO, 2017). In addition to WHO interventions, other psychological treatments have been shown to be effective in alleviating psychological symptoms in asylum seekers and refugees (Uphoff *et al*., 2020; Turrini *et al*., 2021). Notably, a preventative effect on the development of mental disorders has recently been demonstrated for Self Help Plus (SH+), a WHO self-help psychological intervention that can be delivered to up to 30 people at once by briefly trained non-specialist facilitators (Epping-Jordan *et al*., 2016; WHO, 2021*a*). Two randomised trials, one conducted in Western Europe and another in Turkey, have recently assessed the preventative effect of SH+ in refugee populations (Purgato *et al*., 2021; Acarturk *et al*., 2022), while a trial in Uganda showed its beneficial effects for reducing psychological distress (Tol *et al*., 2020). Both the Western European and Turkey studies showed evidence of an effect of SH+ in preventing the onset of mental disorders and reducing stress, but differences were observed between the studies. The effect was much more pronounced for the Turkey study where efficacy (i.e. reducing the frequency of any mental disorder) was observed at 6 months, compared to the Western European study where beneficial effects were only found immediate post-intervention and not after six months (Purgato *et al*., 2021; Acarturk *et al*., 2022). As SH+ is a low-intensity, brief intervention, it cannot deal with the full range of difficulties that refugees and asylum seekers may experience following war-related traumatic events. Therefore, it may be used to complement other psychological or pharmacological interventions, or in addition to psychosocial supports and other needed interventions to ensure stability and safety (IASC, 2007). SH+ was originally devised for administration in a group setting but a component of the course – an illustrated stress management guide with accompanying brief audios of exercises, called ‘Doing What Matters in Times of Stress (DWM)’ – is also available for use as an individually guided or unguided self-help intervention (WHO, 2021*b*). Whilst there has been limited empirical research into DWM, its format is consistent with WHO recommendations for the use of self-help psychological interventions for depression and non-specialist delivery of interventions more generally.

As the number of persons in need of protection is likely to substantially increase globally, driven by long-lasting wars as well as by new conflicts such as the Russo-Ukrainian war which broke out recently, national authorities are urged to develop reception and resettlement programs meeting the needs of vulnerable groups. Evidence-based psychological support should be an important component of such reception programs, aiming to prevent the deterioration of poor mental health towards full-blown psychiatric conditions.
